# Dataset of an in-use tertiary building collected from a detailed 3D mobile monitoring system and building automation system for indoor and outdoor air temperature analysis

**DOI:** 10.1016/j.dib.2020.105907

**Published:** 2020-06-23

**Authors:** Catalina Giraldo-Soto, Aitor Ekoreka, Ander Barragan, Laurent Mora

**Affiliations:** a ENEDI research group, Department of Thermal Engineering, Faculty of Engineering of Bilbao, University of Basque Country (UPV/EHU), Pl. Ingeniero Torres Quevedo 1, 48013 Bilbao, Spain; b I2M - Institute of Mechanics and Engineering - University of Bordeaux CNRS (UMR 5295), site ENSAM, Esplanade des arts et métiers, 33400 Talence, France

**Keywords:** In-use tertiary building, Indoor air temperature, Outdoor air temperature, Temperature uncertainty, Energy performance of buildings (epb)

## Abstract

A Mobile Monitoring System (MMS) has been designed taking into account the use of technology with high sensor accuracy and the ability to be installed easily and quickly in different cardinal locations, distribution spaces, volumes and at different heights of a tertiary in-use building located in Leioa (Bilbao). Two types of MMS have been designed with the objective of carrying out two types of analysis; one intended to do a global indoor air temperature uncertainty analysis and the other focused on doing a global outdoor air temperature uncertainty analysis.

Eight tripods make up the interior MMS with twenty sensors at different heights, which have been installed in different offices in the building to collect indoor air temperature measurements at different heights and locations. In addition, eight sensors make up the exterior MMS to collect data from outdoor air temperature measurements around the building envelope. Both MMS have been integrated into the existing Building Automation System (BAS) of the tertiary building; some other data collected by the BAS has also been taken into account for the uncertainty analysis of indoor and outdoor air temperature.

The interior and exterior MMS datasets have been compiled based on a rigorous data collection process, with the potential to use the data to study the spatial air temperature behavior, taking into account the impact of solar radiation, the heating system and the electrical energy consumption. Furthermore, it enables the global uncertainty of indoor and outdoor air temperature measurements on an in-use building to be estimated and to break it down into the different uncertainty sources, such as the sensor accuracy, vertical and horizontal temperature variability, solar radiation, occupancy and heating system effects. Finally, it enables the optimization of monitoring and control systems for BAS, heating and HVAC systems, as well as any monitoring system implemented in research tests using indoor and/or outdoor temperature measurements as key variables.

**Dataset specifications**

**Table:** 

**Subject**	Energy. (Renewable Energy, Sustainability and the Environment).
**Specific subject area**	Indoor and outdoor air temperature uncertainty analysis of in-use tertiary buildings.
**Type of data**	Tables, text files
**How data were acquired**	Most of the data were collected from a Mobile Monitoring System (MMS), using Modbus [1] RTU-RS485 technology. The rest were acquired from a Building Automation System (BAS) installed during the European Union project: “Affordable and Adaptable Public Buildings through Energy Efficient Retrofitting (A2PBEER)” [2], with KNX [3] technology. MMS is a temporary system, which was integrated into the existing BAS of the tertiary building.
**Data format**	Raw
**Parameters for data collection**	Interior MMS: Sensors were located at different heights and volume distributions. The mobile system is easy to move, connect and install in multiple, different spaces.
	Exterior MMS: Sensors were installed at different heights and cardinal locations around the tertiary building envelope.
	Existing BAS of building: Monitoring System (MS) whose sensors are fixed for the continuous monitoring of the building.
**Description of data collection**	The collected MMS data are the values sent from each sensor and recorded by BAS data acquisition system. The frequency and time instants for data acquisition have been determined by the moment in which a sensor sends a value and this value is recorded. Therefore, not all data columns have the same length.
	Interior MMS were installed in four different offices in the same building in different weeks. The monitoring period for each office test is approximately fifteen days in order to guarantee that enough data is collected without erroneous values for a proper statistical uncertainty analysis of the studied temperature.
	Exterior MMS installed around the tertiary building with different cardinal directions and different heights to collect data during various seasons.
	Likewise, some BAS measurements are included on the data files.
**Data source location**	The west block of the admin building of the University of the Basque Country (UPV/EHU). City/Town/Region: Leioa / Bizkaia / Basque Country. Country: Spain.
	Latitude and longitude (and GPS coordinates): (43.3316308, −2.9716170).
**Data accessibility**	Data are hosted in a public repository: Repository name: Mendeley Data:
	Data identification number: DOI: 10.17632/fc2r9rdxbt.3
	Direct URL to data: https://data.mendeley.com/datasets/fc2r9rdxbt/3

**Value of the data**

• Indoor and outdoor air temperatures represent two of the main variables considered in buildings HVAC system control, to characterize the in-use building envelope energy behavior and to estimate the building energy demand, the users comfort level, among other. The presented datasets permit to study the total uncertainty of indoor and outdoor air temperature, being usual to consider only the sensor accuracy as the measurement uncertainty.
• The scientific community or professionals working on the design and optimization of HVAC system controls in buildings, and on the improvement the building monitoring and controlling systems can use data to improve their researches and products.
• For future research based on smart cities, the datasets of the experimental test carried out allow to improve the energy efficiency of buildings knowing a good representative value of the indoor and outdoor air temperatures to optimize the monitoring and controls of a BAS, HVAC system as well as any monitoring system. It also opens the study to improve the air temperature sensor technology so they can have more accurate and precise sensors.
• The datasets allow complementing the current research studies in order to gain a better knowledge of the energy behavior of buildings and their subsystems, together with the user comfort, to reduce the energy consumption and its impact on the CO2 emissions.
• These data sets make it possible to identify the best locations for indoor and outdoor air temperature sensors, to determine the minimum number of sensors, to estimate the experimental accuracy of the sensor technology and to study the impact of using solar radiation shielding, with and without forced ventilation in outdoor air sensors. All this helps to improve the technology used in monitoring and controlling systems of buildings.
• Collected data allow knowing the representative value of indoor and outdoor air temperature, taking into account the vertical and horizontal stratification by zone or space, the accuracy and precision of sensors. It also provides information on the spatial air temperature as a function of location and number of measurements, the impact of ON/OFF solar radiation, the ON/OFF heating system and the ON/OFF electricity energy consumption.

## 1 Data description

There are three types of dataset; data collected from the existing Building Automation System (BAS) and from two Mobile Monitoring Systems (MMS), interior and exterior MMS. Each system is made up of different technologies.

For the interior experimental test, the Monitoring System (MS) has been conceived to be a mobile system, so as to be able to quickly change the MS to different spaces and floors, adapting it to different distances, heights and geometrics in each space. Eight tripods, twenty sensors, two gateways, Modbus wire and aero-connectors make up the interior MMS. In the case of the MS for the exterior experimental test, eight sensors have been placed around the building at different heights and cardinal orientations. A gateway, Modbus wire and aero-connectors composed the exterior MMS.

The technologies used for the interior and exterior MMS and the existing BAS of the tertiary building are:

upperRoman%1 **Mobile Monitoring System (MMS) for interior measurement composed of:**
  1 Tripods: Eight units.
  2 Sensors:
    a Temperature, relative humidity and Carbon Dioxide (CO_2_): EE800-M12J3 (*E* + *E* Elektronik) [4]. Protocol communication Modbus-RTUS485. Eighteen units.
    b Temperature and relative humidity: EE071-HTPC with shielding (*E* + *E* Elektronik) [5]. Protocol communication Modbus-RTUS485. One unit.
    c Radiant temperature: WBGT-PT100 (4 L) (Ahlborn) [6]. Analogical communication - resistive signal. One unit.
  3 Gateway:
    a Modbus - KNX: KNXRTU1K (DEEI) [7]. Maximum number of points 1000. Supports Boolean data, 8 bits, 16 bits, 32 bits, 64 bits, float 16, float 32. 120-ohm resistor inside the gateway. One unit.
  4 Data collector:
    a Analogical communication: Almemo 2590 (Ahlborn) [8]. One unit.
  5 Power supply:
    a Output 24 V - 4.2A: HDR-100–24 N (Mean Well). One unit.
    b Output 5 V - 3A: HDR-30–5 (Mean Well). One unit.
upperRoman%1 **Mobile Monitoring System (MMS) for exterior measurement composed of:**
  1 Sensors:
    a Temperature and relative humidity: EE071-HTPC with shielding (*E* + *E* Elektronik). Protocol communication Modbus-RTUS485. Eight units.
      • With radiation shielding, but without mechanical ventilation: seven units.
      • With radiation shielding and mechanical ventilation: one unit.
  2 Gateway:
    a Modbus - KNX: IBOX-KNX-MBM (IntsisBox) [9]. One unit.
  3 Power supplies:
    a Output 24 V - 4.2A: HDR-100–24 N (Mean Well). One unit.
    b Output 5 V - 3A: HDR-30–5 (Mean Well). One unit.
upperRoman%1 **The existing Building Automation System (BAS) of the Leioa building:**
  1 Composed of sensors with KNX protocol communication, gateways and power supplies installed by the A2PBEER project of the European Union [2], which are:
    a Sensors (KNX):
      • Electricity meters: EM/S3.16.1 and A43–211 (ABB).
      • Calorimeters: Multical 602 (Kampstrup).
      • Indoor comfort measurements: Temperature, relative humidity and carbon oxide: SK04-S8-CO_2_-TF (ARCUS-EDS).
      • Exterior variable measurements: Temperature and relative humidity: SK01-TFK-AFF and SK10-THC—CO2-KF (ARCUS-EDS).
      • Weather station: SK08-GLBS (ARCUS-EDS).
      • Horizontal global solar radiation: SK08-GLBS (ARCUS-EDS).
    b Power supply: 2005 REG (JUNG). 320 mA.
    c IP gateway: KNX IP Interface 730 (WEINZIERL).
    d Modules: ZS/S1.1 Meter interface (ABB).
  2 Web server [10]:
a Hardware: CBSE Evolution Server (IPAS). Intel N2930, 4 × 1.83 GHz, 4 GB RAM, 128 GB SSD, fanless, <18 Watt, 1x Ethernet, VGA and HDMI.
b Software: IPAS visualization software based on HTML technology (IPAS).

For the experimental test, the dataset collected from the existing BAS of the in-use building consisted of data from some selected electricity meters (EM/S3.16.1), calorimeters (Multical 602) and the Horizontal Global Solar Radiation (SK08-GLBS (ARCUS-EDS)) sensors.

The structure of the data files of the 3D MMS is divided into weeks and per unit of measurement. To identify the datasets for the experimental test,

Table 1 (further details in Section 4.2.1),

**Table 1:** Interior MMS codes: file name codes, file column code and sensor reference.

File Name Codes of Interior Mobile Monitoring System (MMS)					
OT#.T#.H.ID#_ XY(SI)_w# // TT#.T#.H.ID#_ XY(SI)_w#					
File Colum Codes of Interior Mobile Monitoring System (MMS)					
OT#.T#.H.ID#_ XY(SI) // TT#.T#.H.ID#_ XY(SI)					
Codes of Interior MMS Sensor (Sensor Reference)					
T#.H.ID#					
Office Typology (OT) and Tripod Together (TT) Test⁎⁎⁎ (OT#)/ (TT)	Tripod Reference (T#)	Height Location (H)	ID Sensor (ID#)	Sensor Measurement (XY)* and its International System Unit (SI)⁎⁎ (XY(SI))	Week of Year (w#)
OT1	T1	h	1	T(C)	w#
OT2					
OT3	T1	m	2		
OT4	T1	l	3		
TT	T2	h	4		
	T2	m	5		
	T2	l	6		
	T3	h	7		
	T4	h	8		
	T4	m	9		
	T4	l	10		
	T5	l	11		
	T6	h	12		
	T6	m	13		
	T6	l	14		
	T7	h	15		
	T7	m	30	Trad(C)	
	T7	l	16	T(C)	
	T8	l	17		
	T8	h	19		
	T8	m	18		

⁎ Sensor measurement: Air Temperature (T) and Radiant Temperature (Trad).

⁎⁎ SI: Symbol (C) represents Degrees Celsius [ °C].

⁎⁎⁎ The OT test is the information collected from the interior MMS installed in four offices, where each tripod has been distributed in different locations within the monitored office. The TT test is the information collected from interior MMS where the tripods have been situated in the same place so that the twenty sensors have been measuring together at the same height.

Table 2 (further details in Section 4.2.2) and

**Table 2:** Exterior MMS codes: file name codes, file column code and sensor reference.

File Name Codes of Exterior Mobile Monitoring System (MMS)						
E.F#.CO.ID#_ XY(SI)_w# // E.R#.CO.ID#_ XY(SI)_w# // ET.R3.CO.ID#_ XY(SI)_w#						
File Colum Codes of Exterior Mobile Monitoring System (MMS)						
E.F#.CO.ID#_ XY(SI) // E.R#.CO.ID#_ XY(SI) // /ET.R3.CO.ID#_ XY(SI)						
Codes of Exterior MMS Sensor (Sensor Reference)						
F#.CO.ID# // R#.CO.ID#						
Exterior (E) and Exterior Together (ET) Test⁎⁎⁎	Façade (F) / Roof (R)	Floor (#)	Cardinal orientation (CO)	Sensor ID (ID#)	Sensor Measurement (XY)* and its International System Unit (SI)⁎⁎ (XY(SI))	Week of year (w#)
	Location (F/R)					
	F	1	n	20	T(C)	w#
	F	1	n	21		
	F	1	w	22		
	F	1	s	23		
E	F	2	s	24		
ET	R	3	s	25		
	R	3	s	26		
	R	3	n	27		

⁎ Sensor measurement: Air Temperature (T).

⁎⁎ SI: (C) represent Degrees Celsius [ °C].

⁎⁎⁎ The E test is the information collected from the exterior MMS installed around the building envelope; the ET test is the information collected from exterior MMS where the eight sensors have been measuring together at the same place on the roof of the building.

Table 3 (further details in Section 4.1) show the coding for the interior and exterior MMS sensors and the selected measurements of the existing BAS of the in-use building. Table 7,

**Table 3:** Existing BAS codes: file name codes, file column code and sensor reference.

File Name Codes of existing Building Automation System (BAS)					
OT#.T9.m.ID#_ XY(SI)_w# // E.T9.m.ID#_ XY(SI)_w#					
File Column Codes of existing Building Automation System (BAS)					
OT#.T9.m.ID#_ XY(SI) // E.T9.m.ID#_ XY(SI)					
Codes of BAS Sensor (Sensor Reference)					
T9.m.ID#					
Office Typology (OT#) Test / Exterior (E) Test	Virtual Tripod Reference (T#)	Virtual Height location (H)	ID sensor (ID#)⁎⁎⁎	Sensor Measurement (XY)* and its International System Unit (SI)⁎⁎ (XY(SI))	Week of year (w#)
OT1	T9	m	132	pH(W)	w#
OT2			133	pH(W)	
OT3			142	pH(W)	
OT4			143	pH(W)	
E			142,143	pH(W)	
			131	pw(W)	
			141	pw(W)	
			1413	rad(W/m2) or rad(W-m2)	

All data are supplied by the existing BAS.

⁎ Sensor measurement: Heating Power (pH) from calorimeter, Active Power (pw) from electricity meters, Solar Radiation (rad).

⁎⁎ SI: W represents Watts [W] and m2 represents Square Meter [m^2^]. W-m2 and W/m2 represent Watt per square meter [W/m^2^].

⁎⁎⁎ ID sensors: 131 is the total electric power on F2 (F2 is composed of OT1, OT2 and OT3). 141 is the total electric power on F3 (OT4). 132 is the power supplied by the heating system to the north oriented offices at F2 (OT1 and OT2). 133 is the power supplied by the heating system to the south oriented offices at F2 (OT3). 142 is the power supplied by the heating system to the north oriented areas at F3 (OT4). 143 is the power supplied by the heating system to the south oriented areas at F3 (OT4). 142,143 is the total heating power supplied by the heating system to the F3 (sum of 142 and 143). 1413 is the horizontal global solar radiation. The measurement of 1413 is the only data that have been taken into account in both the OT test and the E test; the rest of the sensor measurements are taken into account only in the OT test, depending on the floor where the test is done.

Table 8 and Table 9 show the sensor reference and location. To identify the MMS sensors in diagrams; Fig. 8,Fig. 9,Fig. 10,Fig. 11 and Fig. 12 show a schematic location of the sensors.

## 2 Experimental design, materials and methods

**Fig. 1:**
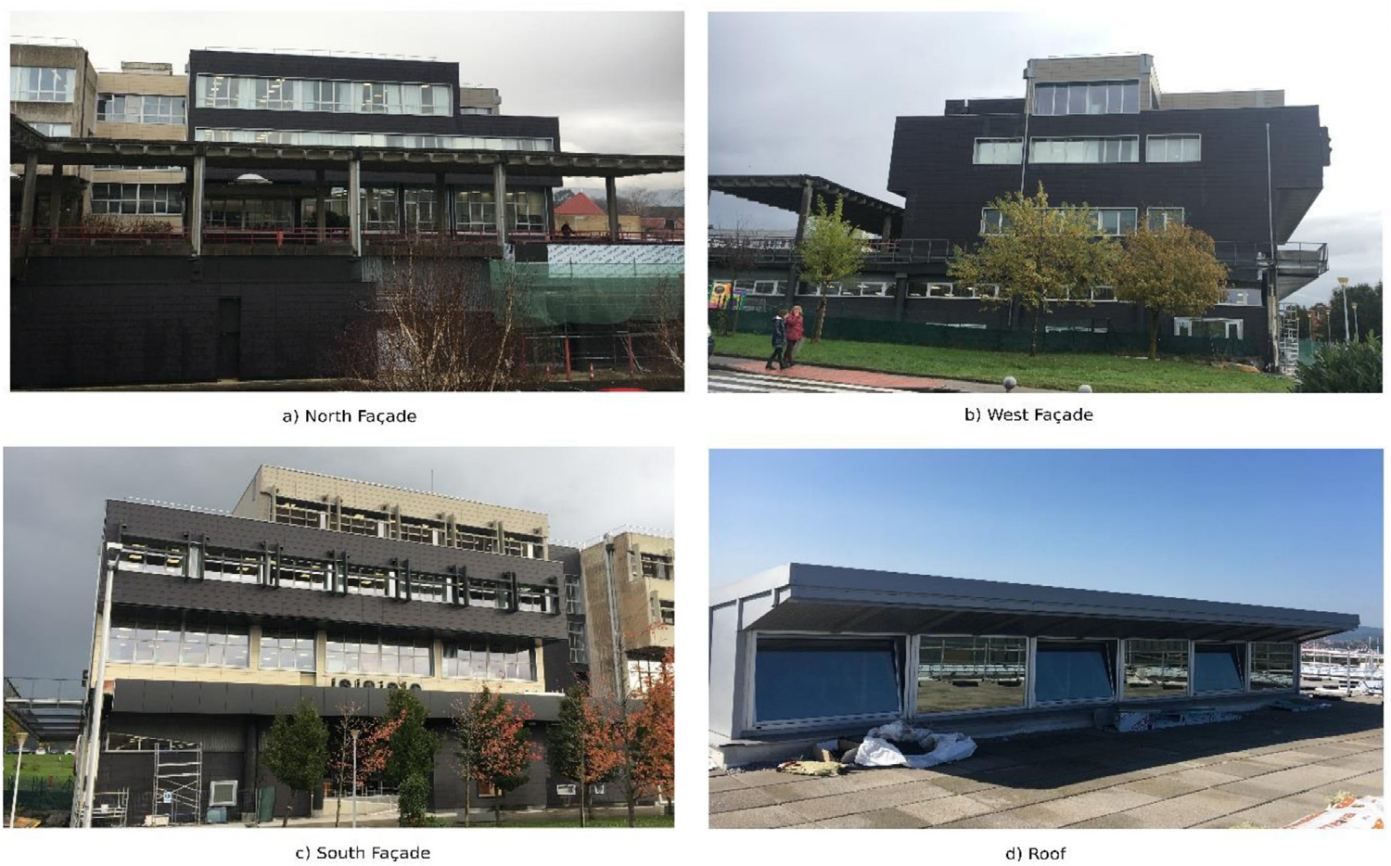
The UPV/EHU administrative building in Leioa post-retrofitting: a) North Façade, b) West Façade, c) South Façade, d) Roof.

The designed Mobile Monitoring System (MMS) was implemented to collect data in spaces with different distributions, cardinal orientations, volumes and at different heights of a tertiary building located in Leioa (Bilbao). The in-use building had been retrofitted in 2018 as a demonstrator building of the project “Affordable and Adaptable Public Buildings through Energy Efficient Retrofitting (A2PBEER)” [2], and was equipped with a BAS system in 2013. The building after retrofitting is shown in Fig. 1.

The following sections describe the building`s characteristics and its existing BAS, along with the description of the interior and exterior MMS. In each section, there is information on the technical specifications, experimental layout distribution and geometric information of each monitored area.

### 2.1 The existing building automation system (BAS) and selected measurements

**Fig. 2:**
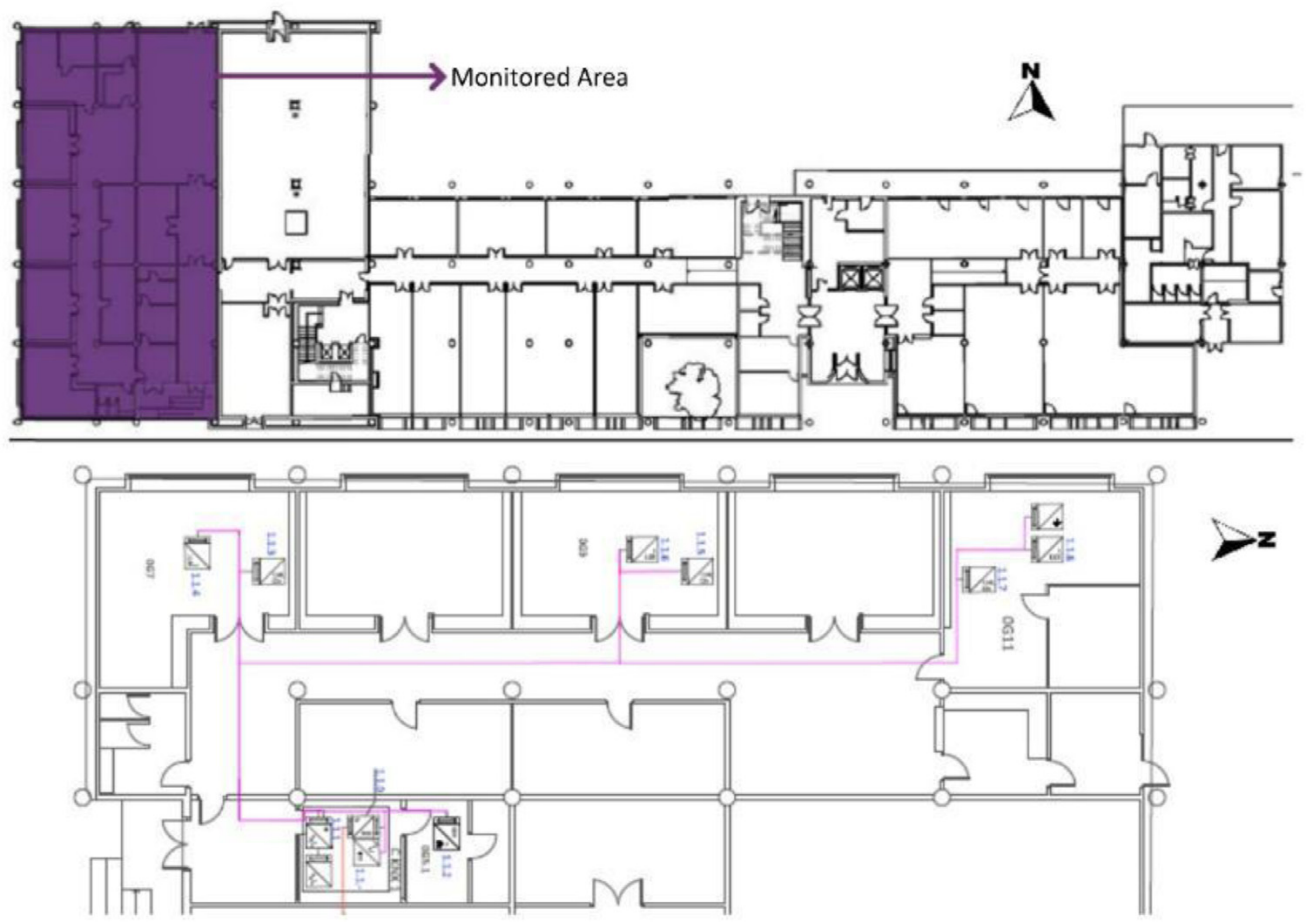
F0 of the UPV/EHU admin building in Leioa. Based on A2PBEER project's architecture plans [2].

**Fig. 3:**
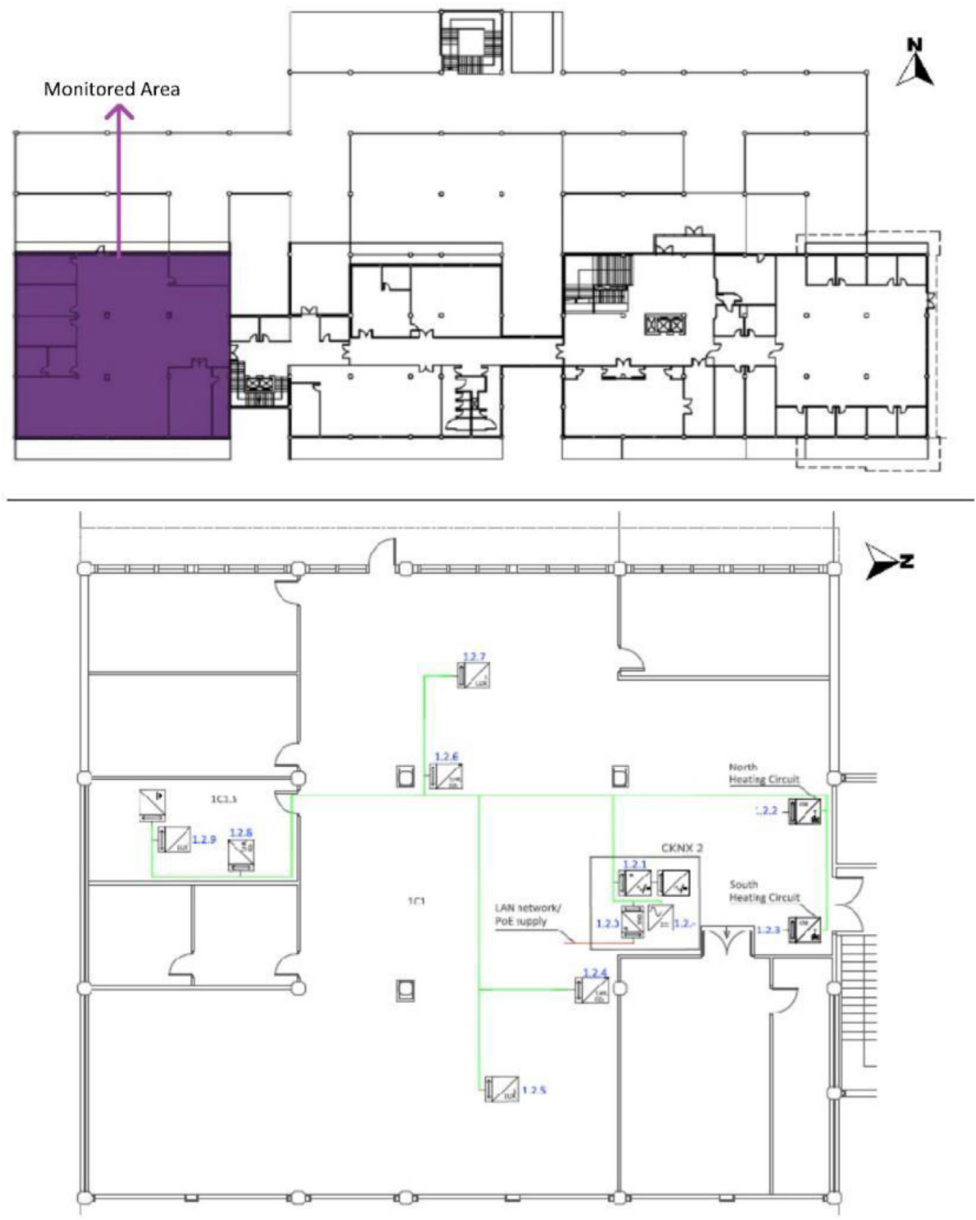
F1 of the UPV/EHU admin building in Leioa. Based on A2PBEER project's architecture plans [2].

**Fig. 4:**
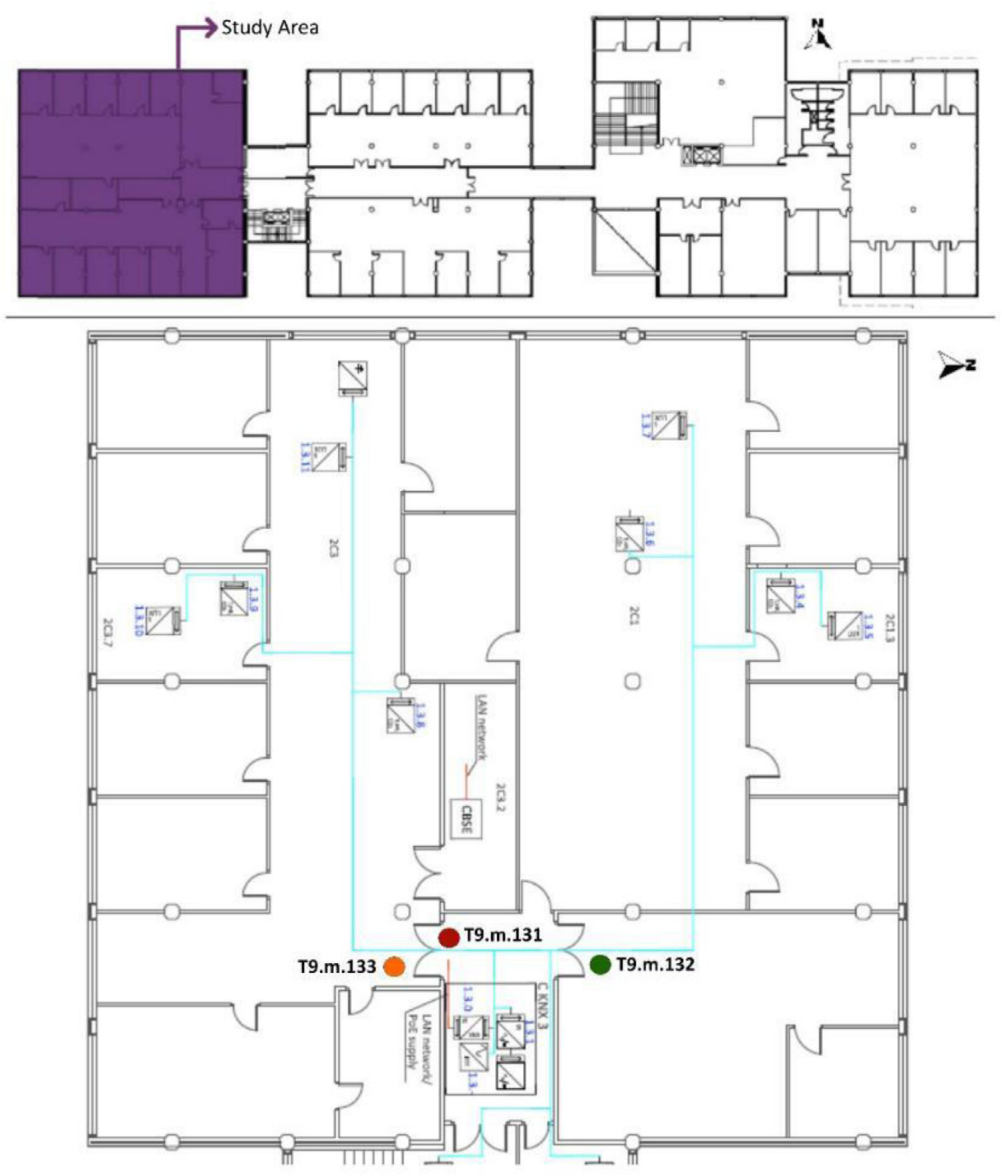
F2 of the UPV/EHU admin building in Leioa. Includes the position of the three selected measurement points of the existing BAS referred to in Table 3. Based on A2PBEER project's architecture plans [2].

**Fig. 5:**
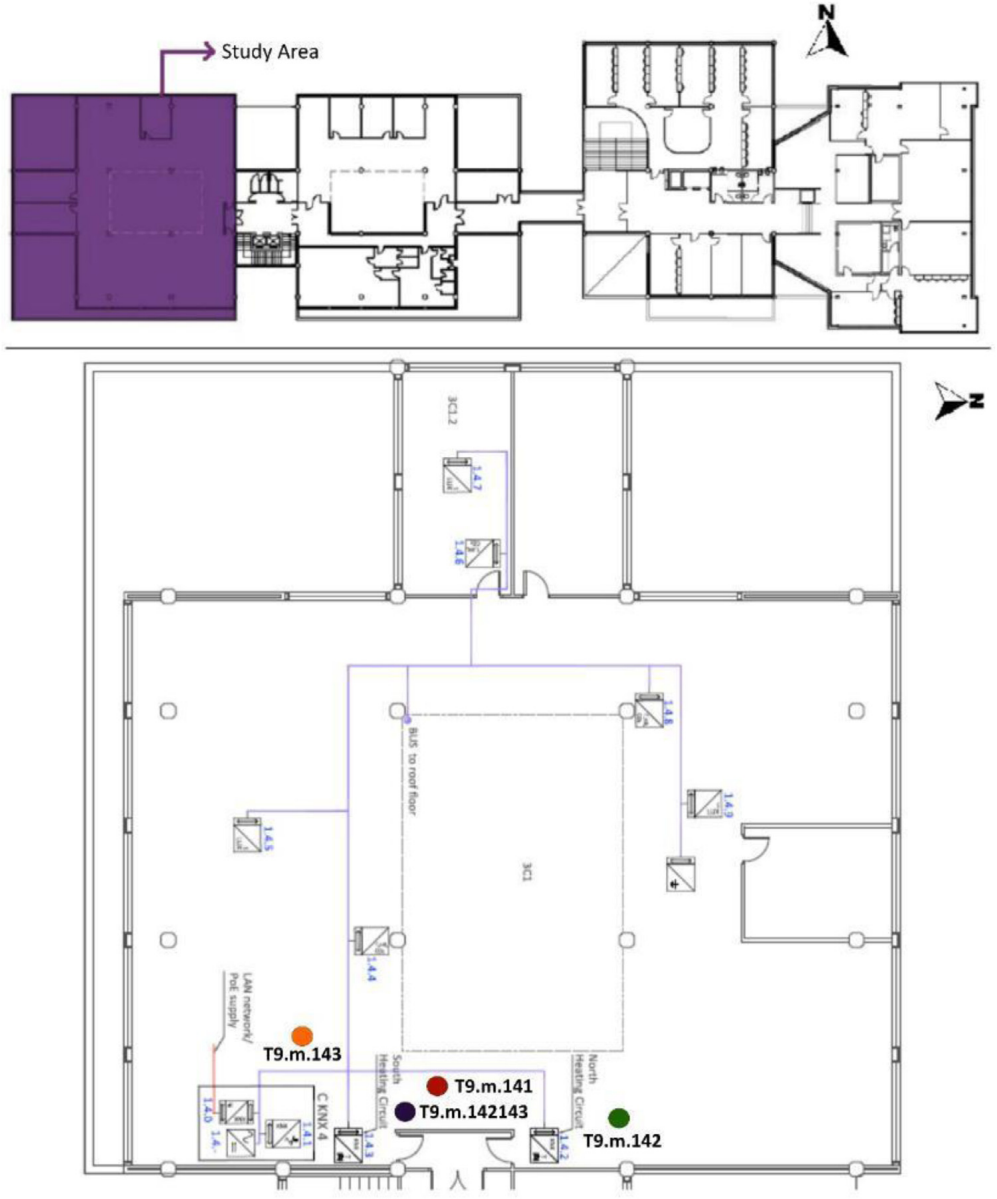
F3 of the UPV/EHU admin building in Leioa, including the position of the three selected measurement points of the existing BAS referred to in Table 3. Note that T9.m.142143 is the signal created by adding the measurements T9.m.142 and T9.m.143. Based on A2PBEER project's architecture plans [2].

**Fig. 6:**
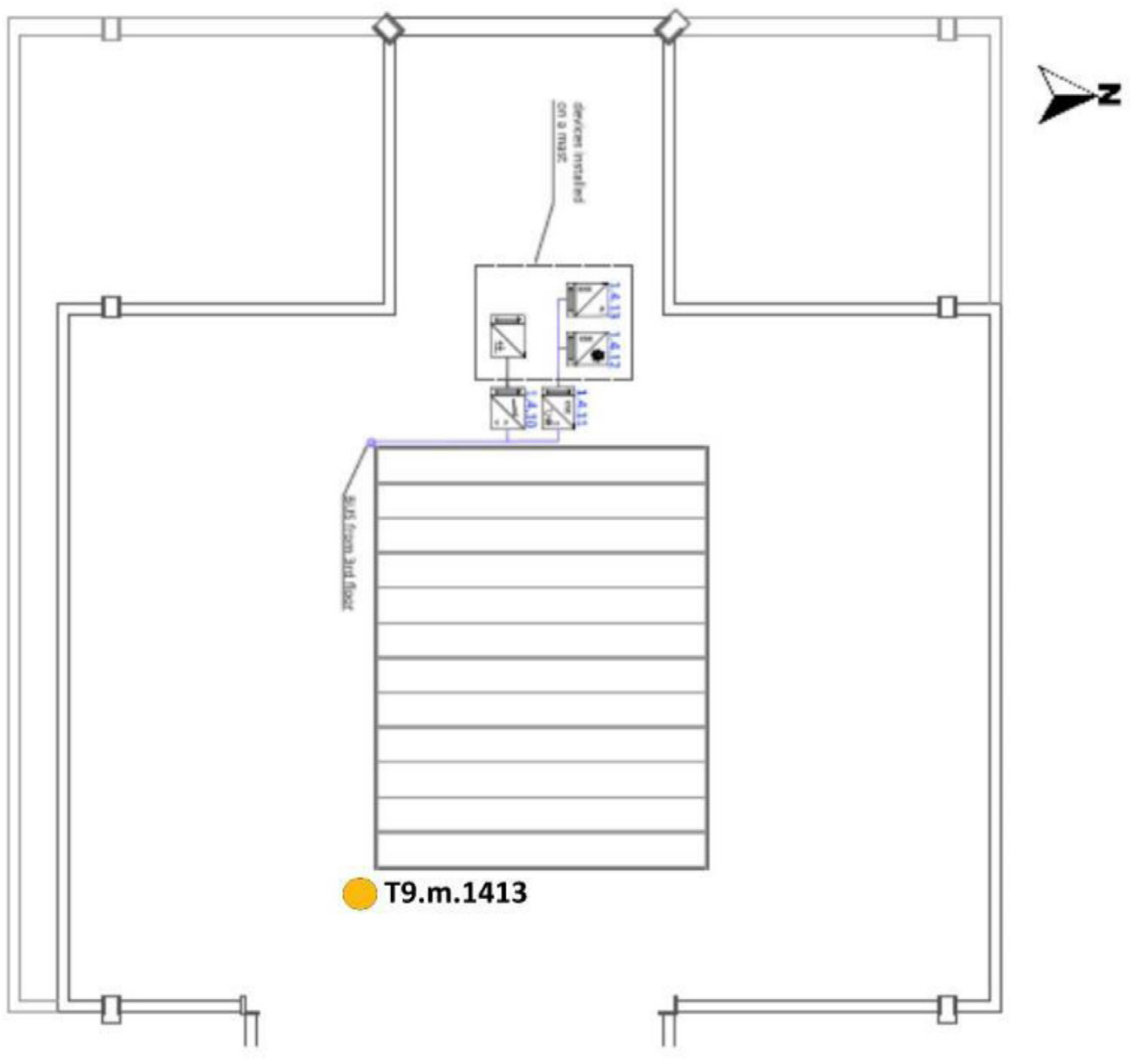
Roof of the UPV/EHU admin building in Leioa, including the position of the selected measurement point of the existing BAS referred to in Table 3. Based on A2PBEER project's architecture plans [2].

The tertiary building studied is the west block of the administrative building of the University of the Basque Country (UPV/EHU) and consists of four floors. A nursery is located on the ground floor (F0) while the other three floors are made up of offices (floor one (F1), floor two (F2) and floor three (F3)). There is currently an existing Building Automation System (BAS) which was implemented during the A2PBEER project, with KNX protocol communication [3]. The KNX sensors installed in the existing BAS are described in Table 4. Fig. 2,Fig. 3,Fig. 4, Fig. 5, Fig. 6 show the floor layouts for each building level, including the roof, and the selected BAS sensor references provided in this document.

**Table 4:** BAS sensors installed at the UPV/EHU administrative building in Leioa: Reference, measurements, accuracies and protocol communication.

Sensor reference	Measure	Accuracy	Protocol Communication
KAMSTRUP: Multical 602	Heat Energy and/or power	ET ± (0.4 + 4/ΔT)%	Digital - KNX
ABB:	a. Lighting power/consumption.	a. ±2%	Digital - KNX
a. A43–211	b. Rest of electricity power/consumption except a.	b. ±1%.	
b. EM/S 3.16.1	Total power/consumption: *a* + *b*.	Total consumption ±3%	
ARCUS:SK04-S8-CO2-TF	Illuminance (lux)	—	Digital - KNX
	Air Quality (ppm CO_2_)	±1% Measurement Error	
	Temperature ( °C)	±0.5 °C	
	Relative Humidity (%)	±3% RH	
ELSNER: 3595 Sun tracer	Illuminance (lux)	±35% at 0…150,000 lx	Digital - KNX
	Temperature ( °C)	±0.5 °C	
	Wind Speed (m/s)	±25% at 0…15 m/s	
	Rain (yes/no)	–	
ARCUS: SK01-TFK-AFF	Temperature ( °C)	±0.5 °C	Digital - KNX
	Relative Humidity (%)	±3% RH	
ARCUS: SK10-THC-CO_2_-KF	Temperature ( °C)	±0.4 °C (5 to 60 °C), else ±0.8 °C	Digital - KNX
	Relative Humidity (%)	±3% RH	
	Air Quality (ppm CO_2_)	±(50 ppm +3% Measurement)	
ARCUS: SK08-GLBS	Horizontal Global Solar Radiation (W/m2)	±5%	Digital - KNX

The MMS experimental test was carried out on two of the four floors of this tertiary building, floors two (F2) and three (F3). These were selected because they can represent four different types of office layouts, each one representing a different office typology. F2 has the particularity that it is made up of three different, independent office spaces and F3 is a single office.

#### 2.1.1 Office typologies (OT)

The offices monitored in this experimental test have different cardinal orientations, distributions, geometry and volumes, each of them with different typologies. Each monitored office will be identified as an Office Typology (OT), where each one has been classified according to the number of internal divisions called workspaces (WS) and each OT is located:

• Office Typology 1 (OT1): Located in F2.
• Office Typology 2 (OT2): Located in F2.
• Office Typology 3 (OT3): Located in F2.
• Office Typology 4 (OT4): Located in F3.

Table 5 shows the areas, heights and volumes of each OT and WS according to the architectural drawings shown in Fig. 8, Fig. 9, Fig. 10 and Fig. 11.

**Table 5:** Areas, heights and volumes of OT and WS based on the architectural drawings shown in Fig. 8, Fig. 9, Fig. 10 and Fig. 11.

Office	WS Reference in drawings	Area (m^2^)	Height (m)	Volume (m^3^)
**OT1**	2C1	126.03	3.39	427.24
	2C1.1	15.94	3.07	48.94
	2C1.2	16.25	3.10	50.38
	2C1.3	16.18	3.11	50.32
	2C1.4	16.25	3.12	50.62
	2C1.5	16.25	3.13	50.86
	2C1.6	18.06	3.16	56.98
**OT2**	2C2	62.45	3.15	196.72
	2C2.1	11.85	3.12	36.97
**OT3**	2C3	110.22	2.95	325.15
	2C3.2	15.97	3.13	49.99
	2C3.3	14.83	2.91	43.08
	2C3.4	30.70	2.98	91.49
	2C3.5	18.60	2.98	55.34
	2C3.6	18.60	2.95	54.87
	2C3.7	18.53	2.93	54.29
	2C3.8	18.60	2.93	54.41
	2C3.9	18.21	2.93	53.26
OT4*	3C1*	400.40	3.55	1472.98
	3C1.1	16.10	3.36	54.02
	3C1.2	23.99	3.36	80.49
	3C1.3	23.99	3.36	80.49

⁎ The 3C1 height shown is a mean value of this WS. Nevertheless, the volume shown takes into account the different heights within this WS. All south façade windows have external shading elements, except in the WS reference 3C1.2. Windows in the north, east and west façades have no shading elements.

#### 2.1.2 Selected datasets from the existing building automation system (BAS)

Some selected datasets measured by the existing Building Automation System (BAS) have been included along with the Mobile Monitoring System (MMS) datasets. The main variables affecting the behavior of the indoor air temperature, such as the heat power input of the heating system, the total electricity active power consumption within the analysed office, and the horizontal global solar radiation, have been included in this document. The last signal is also important if the outdoor air temperature is to be analysed.

Table 3 shows the file name code list for these datasets and a detailed description of the whole existing monitoring system can be found in [11]. Similarly, the position of these selected sensor references, included in the experimental test, are shown in Fig. 2, Fig. 3, Fig. 4, Fig. 5 and Fig. 6.

### 2.2 Description of interior and exterior experimental tests using a 3D mobile monitoring system (MMS)

The criteria for choosing the technology for a monitoring and control system in a BAS or in experimental tests are important to determine the accuracy level of the sensors and their measurements. The technology currently used in domotic systems and BAS do not have the high precision and accuracy of laboratory technology; so it is necessary to introduce technology with greater accuracy and precision in order to increase the reliability of the building monitoring and control systems [12]. Based on this perspective, the technology selected for this experimental test has been chosen with high precision sensors in mind, such as the sensors used in laboratory tests. The selection criteria were:

1 Monitoring technology characterised by
  a High accuracy.
  b Monitoring systems used in industry.
2 Protocol communication:
  a Digital protocol.
  b Frequently used in industrial MCS and not in domotic systems.
  c Protocol that can, in the future, be compared to Transmission Control Protocol/Internet Protocol (TCP/IP)^1^ protocol communication.
3 Hardware:
  a Gateways with a capacity to integrate the new protocol communication and technology in the existing BAS of the tertiary building, which use KNX technology and protocol communication.
4 Viable costs.

In the following subsections, the implemented interior and exterior MMS technology is described, together with the MMS layout.

#### 2.2.1 Interior experimental test and its 3D MMS on the different OT

**Fig. 7:**
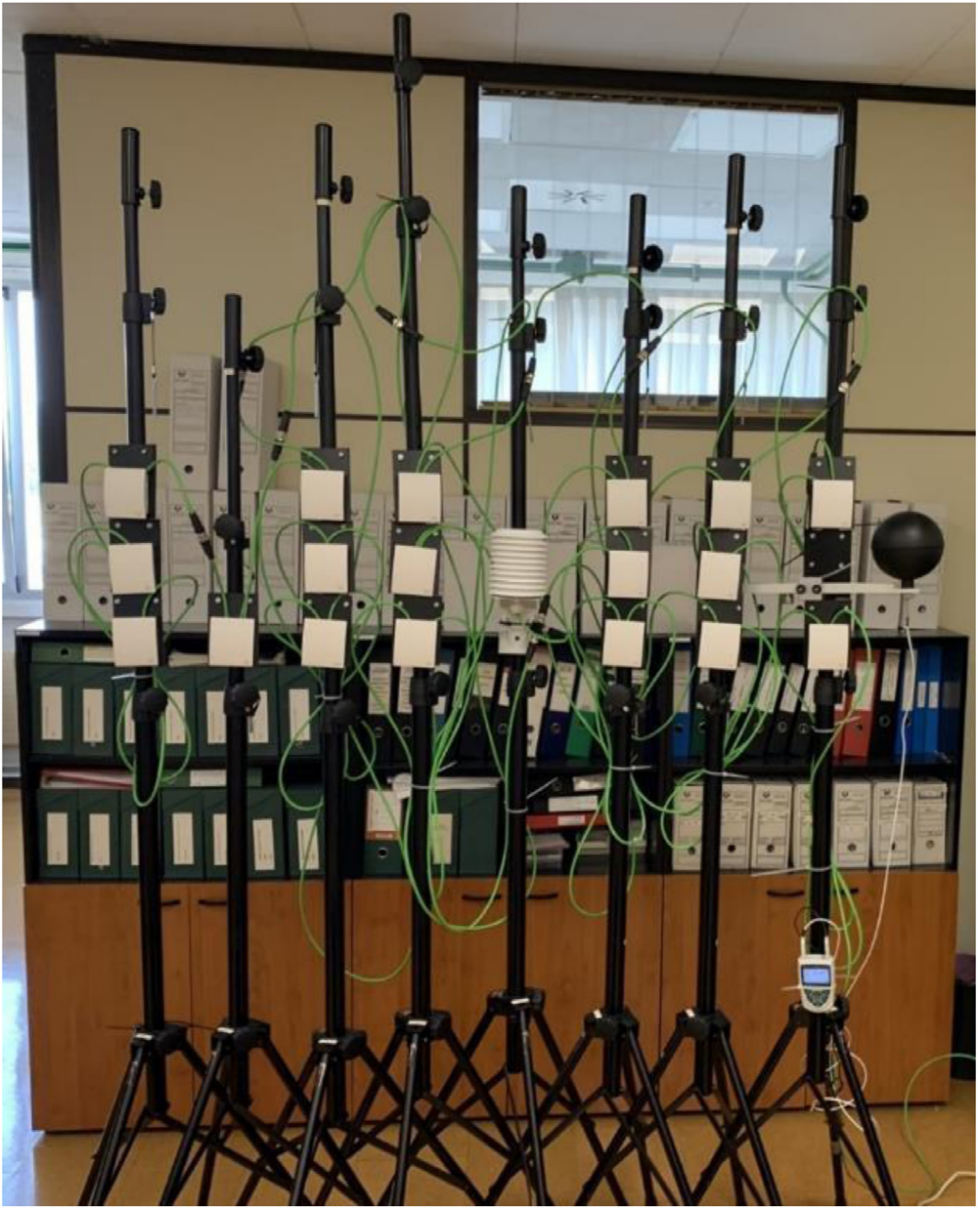
Interior 3D MMS tripods and sensors all together during the TT test.

The Monitoring System (MS) implemented in the experimental test is a mobile system that uses eight tripods distributed in the different volumes of the monitored Office Typologies (OT). Twenty sensors have been installed on eight tripods at different heights (shown in Fig. 7), while the types of sensor and their accuracy is described in Table 6.

**Table 6:** Technical characteristics of sensors, gateway and protocol communications of interior MMS.

Sensor reference	Measure	Accuracy	Protocol communication
EE+Plus: EE800-M12J3	Temperature	± 0.3 °C	Digital - Modbus RS485
	Relative Humidity	± 3% RH(30..70%RH)	
		+/- 5% RH(10..90%RH)	
	Carbon Dioxide	0…2000 ppm < ± (50 ppm +2% of measured value)	
EE+Plus EE071-HTPC	Temperature	±0.1 °C at 23 °C	Digital - Modbus RS485
	Relative Humidity	±2% RH (0…90% RH)	
		±3% RH (0…100% RH)	
Ahlborn: WBGT - PT100 (4 L)	Radiant Temperature	PT 1000 Class B (−50 °C to +200 °C)	Analogic - Resistive

Reference	Producer	Protocols	Description
KNXRTU1K [7]	DEEI	KNX to Modbus RTU-RS485	RS485 Half-Duplex interface for Modbus RTU.
			The 120-ohm RS485 termination resistor inside the gateway.
			Operating temperature −40 to + 85 °C.
			Maximum number of points 1000.
			Supports Boolean data, 8 bits, 16 bits, 32 bits, 64 bits, float 16, float 32.

The protocol communication implemented in the MMS was Modbus RTU-RS485 [1]. For data collection, it was necessary to integrate the MMS into the admin building's BAS, which works with KNX protocol communication. It was necessary to use KNX Modbus RTU- RS485 gateways to integrate the MMS to the existing BAS [1]. The use of these gateways allowed the collected MMS data to be sent to the web server, and all the information to be exported to a single database. The gateway brand used is a DEEI KNX-Modbus RTU, whose reference is KNXRTU1K [7].

Table 6 shows a brief technical description of the installed gateway.

The eight tripods that make up the MMS were distributed spatially and temporally in different OTs of F2 and F3. The tripods were interconnected using aero-connectors with different wire lengths, allowing for a quick installation of the MMS and adaption of the system to the different spatial geometries.

Table 7 shows the position of each sensor on each tripod, as well as sensor and manufacturing references.

**Table 7:** Sensor references installed on the eight tripods and level location.

Sensor Reference	Tripod Number	Height	Sensor ID	Sensor Manufacture Reference
T1.h.1	T1	h	1	EE800-M12J3
T1.m.2		m	2	EE800-M12J3
T1.l.3		l	3	EE800-M12J3
T2.h.4	T2	h	4	EE800-M12J3
T2.m.5		m	5	EE800-M12J3
T2.l.6		l	6	EE800-M12J3
T3.h.7	T3	h	7	EE800-M12J3
T4.h.8	T4	h	8	EE800-M12J3
T4.m.9		m	9	EE800-M12J3
T4.l.10		l	10	EE800-M12J3
T5.l.11	T5	l	11	EE800-M12J3
T6.h.12	T6	h	12	EE800-M12J3
T6.m.13		m	13	EE800-M12J3
T6.l.14		l	14	EE800-M12J3
T7.h.15	T7	h	15	EE800-M12J3
T7.m.30		m	30	WBGT - PT100
T7.l.16		l	16	EE800-M12J3
T8.h.17	T8	l	17	EE800-M12J3
T8.m.19		h	19	EE071-HTPC⁎⁎
T8.l.18		m	18	EE800-M12J3

⁎⁎ EE071-HTP with radiation shielding without mechanical ventilation.

**Fig. 8:**
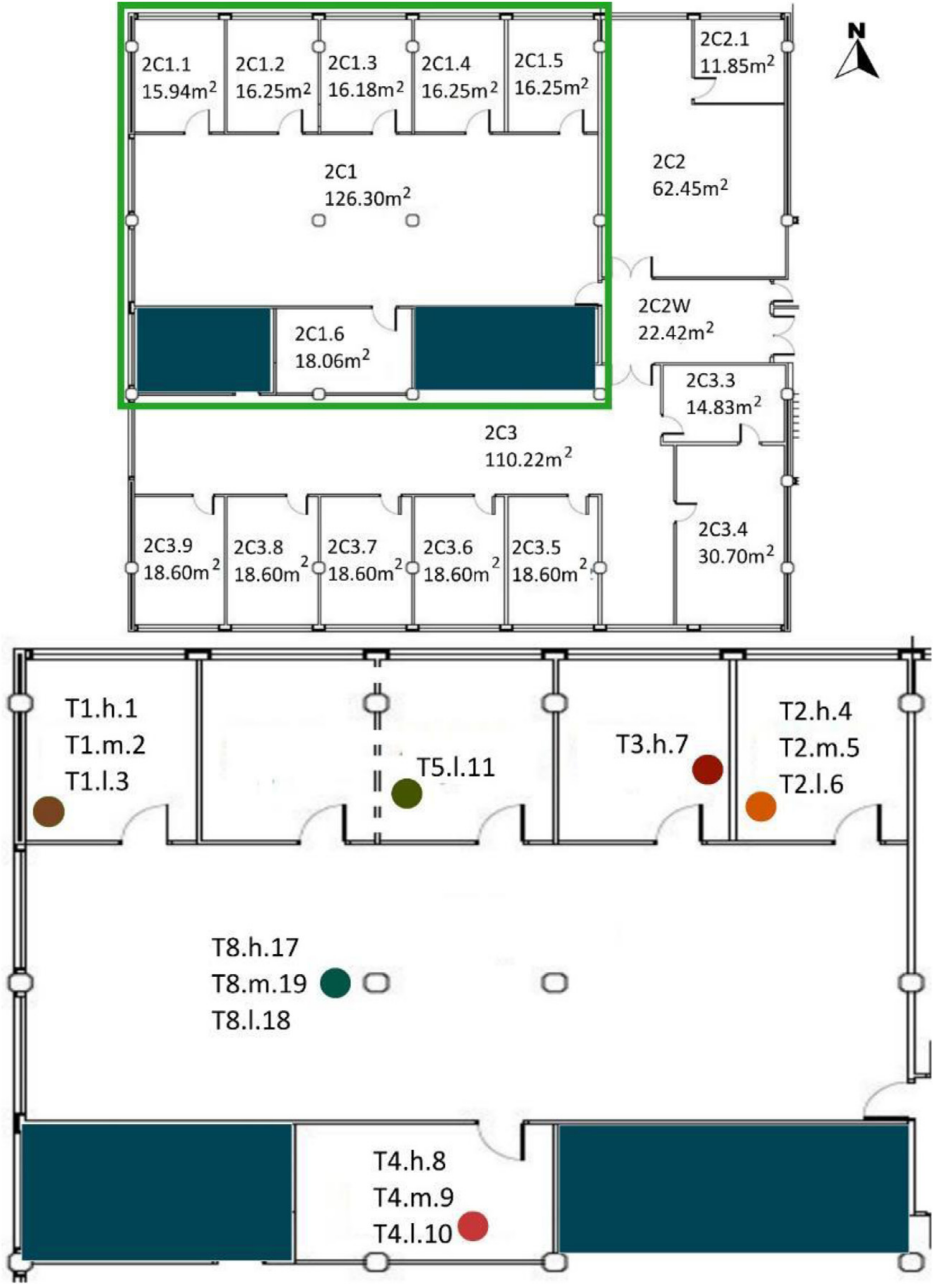
OT1 sensor layout, located in F2. Based on A2PBEER project's architecture plans [2].

**Fig. 9:**
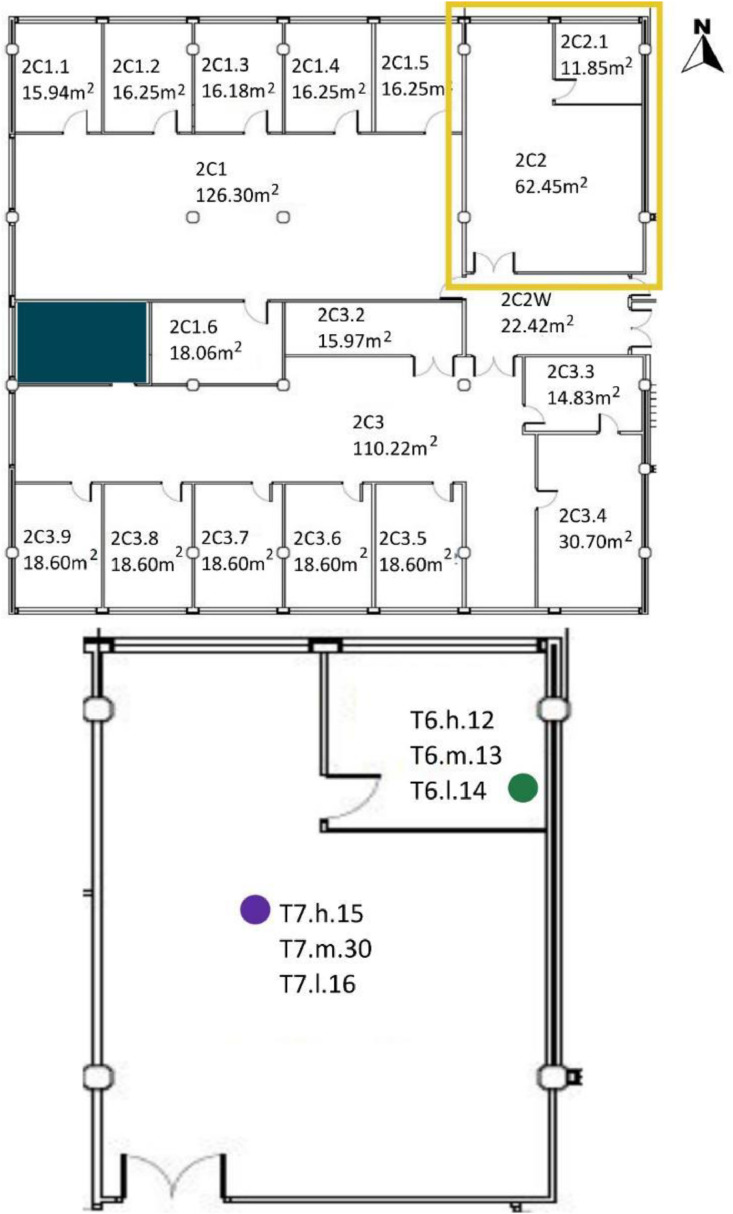
OT2 sensor layout, located in F2. Based on A2PBEER project's architecture plans [2].

**Fig. 10:**
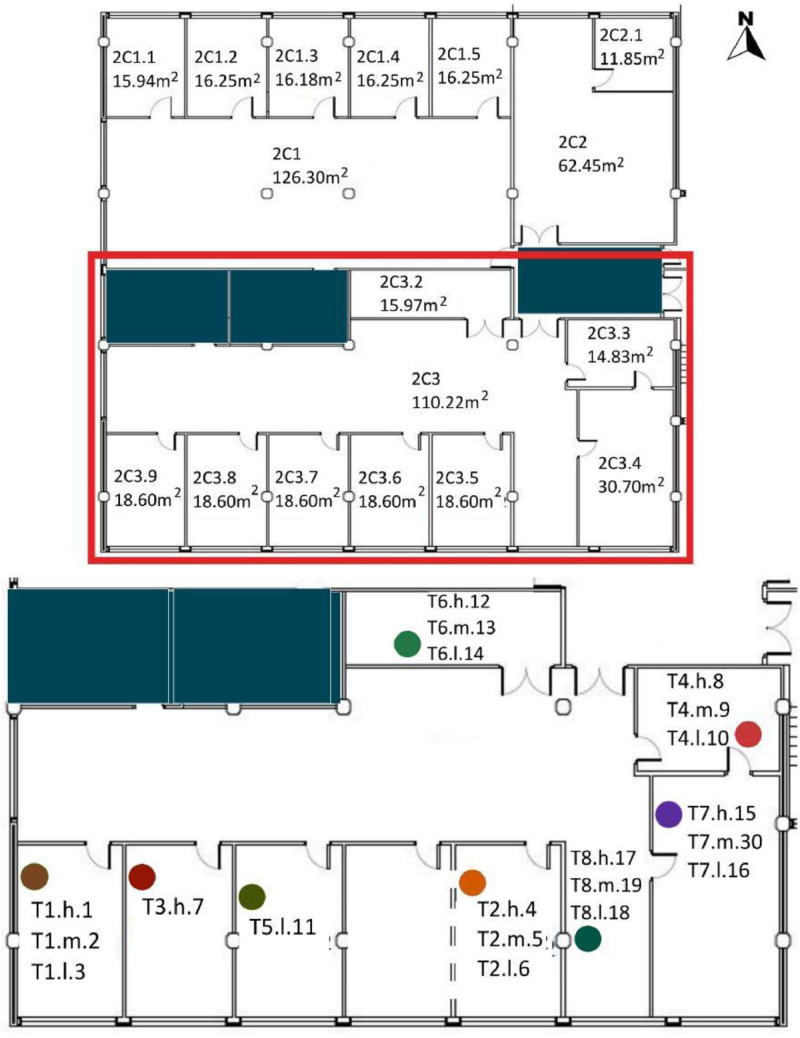
OT3 sensor layout, located in F2. Based on A2PBEER project's architecture plans [2].

**Fig. 11:**
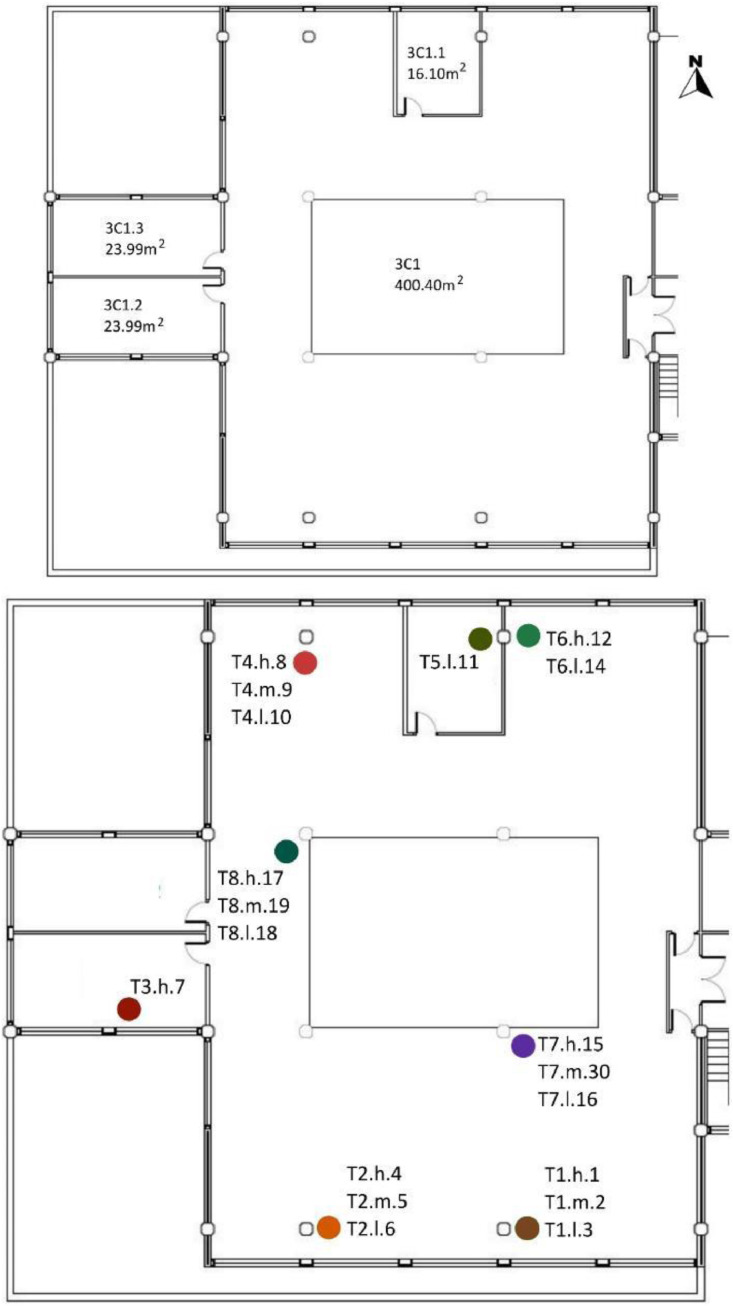
OT4 sensor layout, located in F3. Based on A2PBEER project's architecture plans [2].

Table 8 shows the WS location in each OT with respect to the architectural drawings shown in Fig. 8, Fig. 9, Fig. 10, Fig. 11. The encoding of the dataset files is shown in Table 1.

**Table 8:** Sensor layout by WS in each OT volume.

Office typology	Number of WS	WS Reference	Sensor Reference
**OT1**	**6**	2C1	T8.h.17 - T8.m.19 - T8.l.18
		2C1.1	T1.h.1 - T1.m.2 - T1.l.3
		2C1.2	No tripod
		2C1.3	T5.l.11
		2C1.4	T3.h.7
		2C1.5	T2.h.4 - T2.m.5 - T2.l.6
		2C1.6	T4.h.8 - T4.m.9 - T4.l.10
**OT2**	**1**	2C2	T7.h.15 - T7.m.30 - T7.l.16
		2C2.1	T6.h.12 - T6.m.13 - T6.l.14
**OT3**	**8**	2C3	T8.h.17 - T8.m.19 - T8.l.18
		2C3.2	T6.h.12 - T6.m.13 - T6.l.14
		2C3.3	T4.h.8 - T4.m.9 - T4.l.10
		2C3.4	T7.h.15 - T7.m.30 - T7.l.16
		2C3.5	T2.h.4 - T2.m.5 - T2.l.6
		2C3.6	No tripod
		2C3.7	T5.l.11
		2C3.8	T3.h.7
		2C3.9	T1.h.1 - T1.m.2 - T1.l.3
**OT4**	**3**	3C1	T1.h.1 - T1.m.2 - T1.l.3 - T2.h.4 - T2.m.5 - T2.l.6 - T4.h.8 - T4.m.9 - T4.l.10 - T6.h.12 - T6.l.14 - T7.h.15 - T7.m.30 - T7.l.16 - T8.h.17 - T8.m.19 - T8.l.18
		3C1.1	T5.l.11
		3C1.2	T3.h.7
		3C1.3	No tripod

Two types of test have been carried out using the interior MMS:

• **Office Typology (OT) test**: The OT test period datasets are prefixed by *OTp.Tj*, with *p* = 1 to 4 and *j* = 1 to 8 (see Table 1). Four office typologies were monitored, OT1, OT2, OT3 and OT4. The sensors were installed at different heights on each tripod:
  - High (h): Located 30 cm from the OT ceiling.
  - Medium (m): Located midway between the ceiling and floor of each OT.
  - Low (l): Located 30 cm from the OT floor.
• **Tripod Together (TT) test**: The TT period test datasets are prefixed by *TT.Tj* with *j* = 1 to 8 (see Table 1). All sensors were installed at the same height (at an average of 174 cm with *a* ± 12 cm strip) and the same location (see Fig. 7).

#### 2.2.2 Exterior experimental test and its 3D MMS

Exterior MMS were located around the building's façade and roof. Eight sensors were located on the Exterior (E) of the building envelope at different heights:

• Façades (F): At F1 height and F2 height.
• Roof (R): At F3 height.

**Fig. 12:**
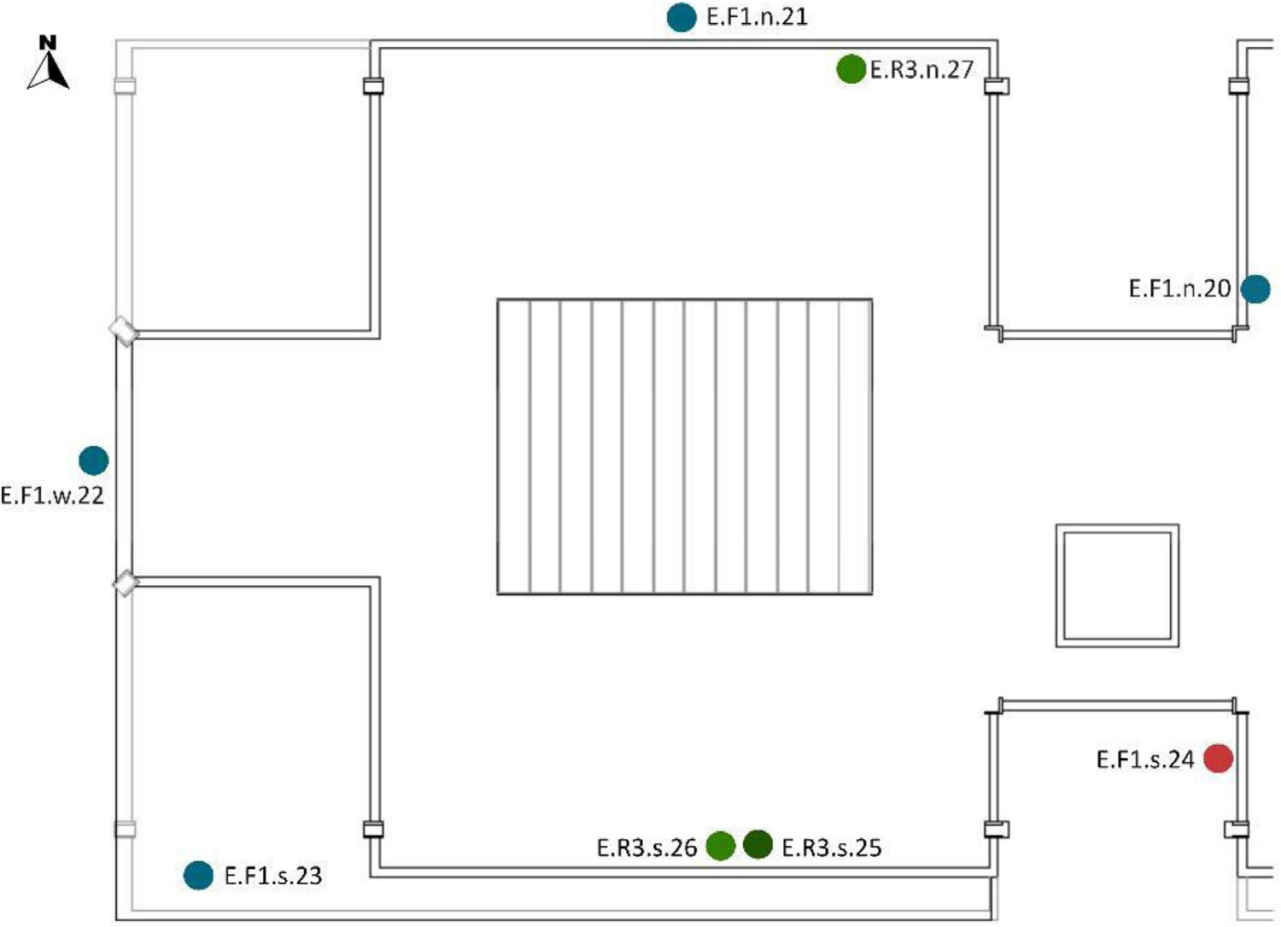
Upper view of the exterior sensor layout around the building envelope. Based on A2PBEER project's architecture drawings [2].

**Fig. 13:**
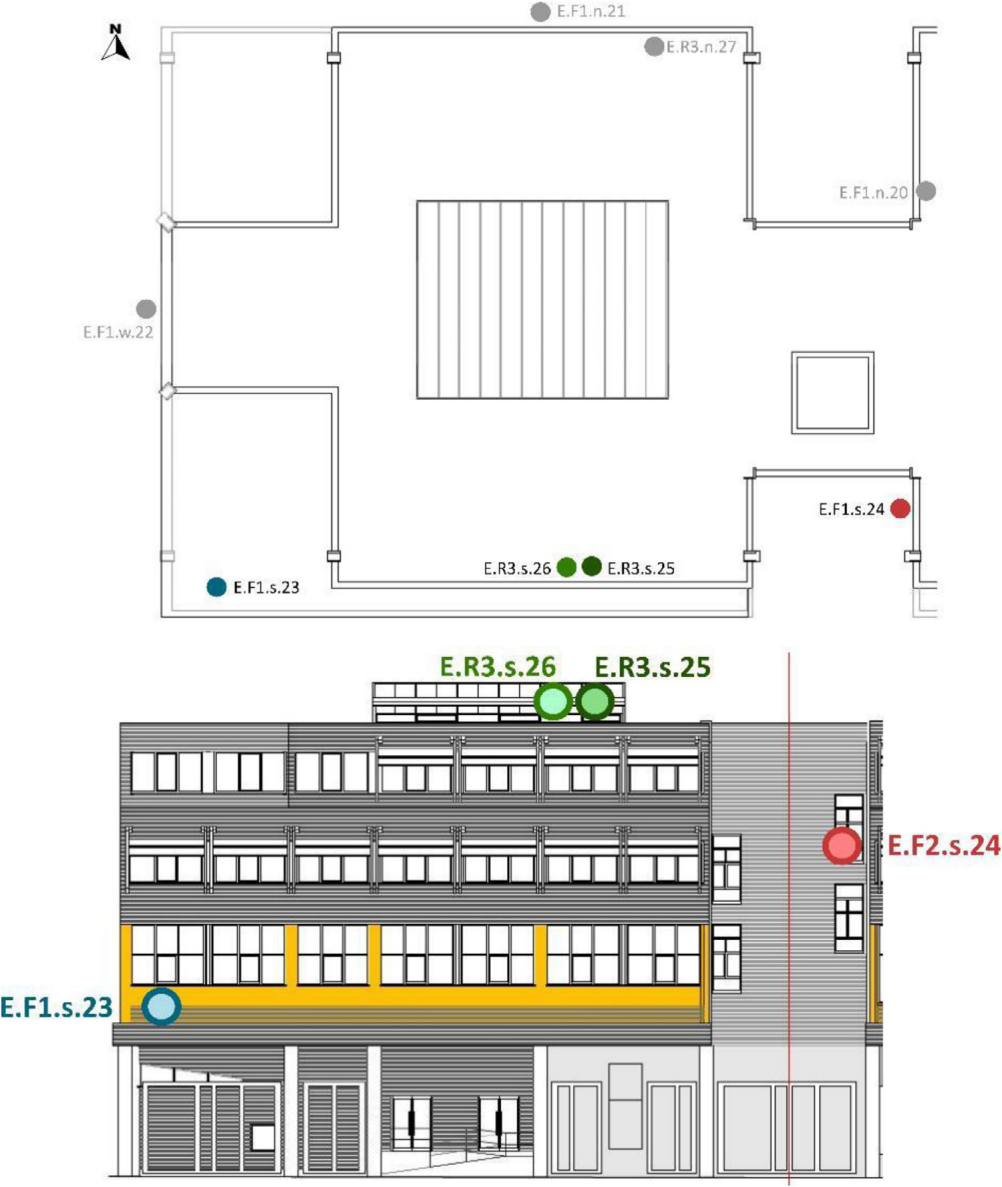
Exterior sensor layout on the south façade of the building. Based on A2PBEER project's architecture drawings [2].

**Fig. 14:**
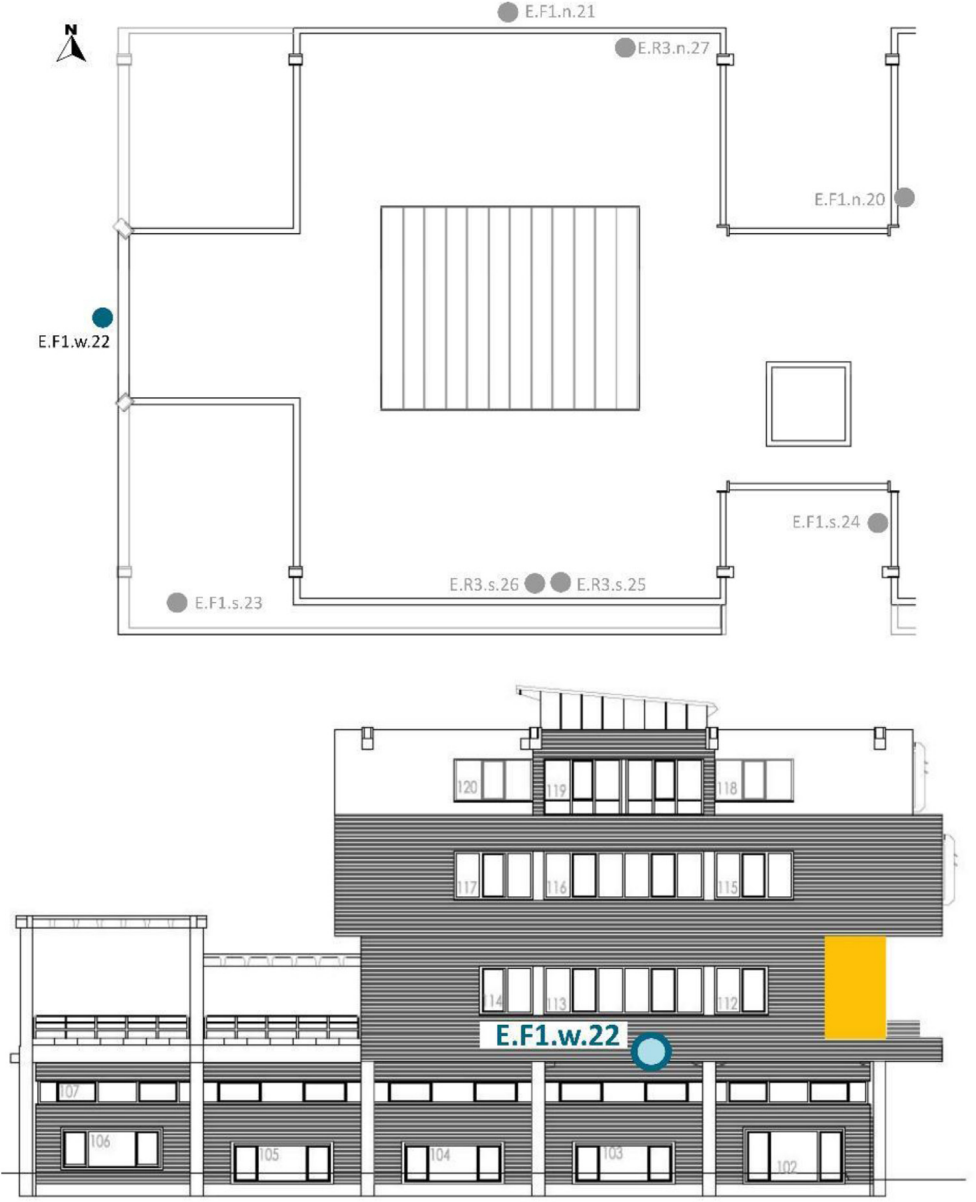
Exterior sensor layout on the west façade of the building. Based on A2PBEER project's architecture drawings [2].

**Fig. 15:**
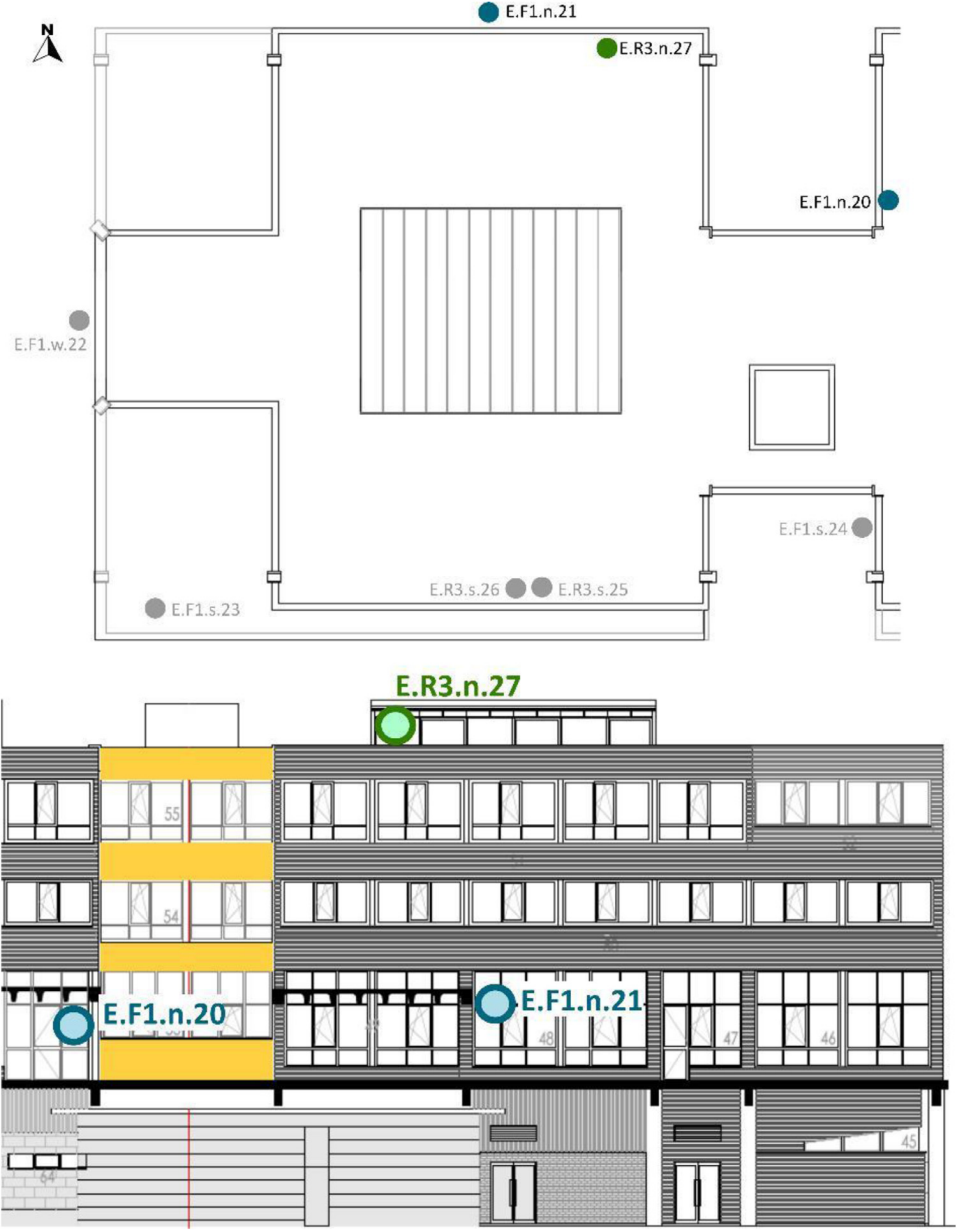
Exterior sensor layout on the north façade of the building. Based on A2PBEER project's architecture drawings [2].

Furthermore, the sensors were located at different cardinal orientations: North (n), South (s), East (e) and West (w). Seven out of the eight installed EE071-HTP sensors were protected against solar radiation using shields without mechanical ventilation and one with mechanical ventilation. Table 9 shows the sensor reference, cardinal orientation and height location of each sensor. Fig. 12, Fig. 13, Fig. 14, Fig. 15 show, in the architectural drawings, the location of each sensor on the building envelope. Remember that these dataset file codifications are presented in Table 2.

**Table 9:** Exterior (E) Layout of EE071-HTPC sensors installed around the building envelope.

Sensor Reference	Façade (F)/Roof (R)	Floor	Cardinal orientation	Sensor ID	Sensor Manufacture Reference
E.F1.n.20	F	1	n	20	EE071-HTP*
E.F1.n.21	F	1	n	21	EE071-HTP*
E.F1.w.22	F	1	w	22	EE071-HTP*
E.F1.s.23	F	1	s	23	EE071HTP*
E.F2.s.24	F	2	s	24	EE071-HTP*
E.R3.s.25	R	3	s	25	EE071-HTP⁎⁎
E.R3.s.26	R	3	s	26	EE071-HTP*
E.R3.n.27	R	3	n	27	EE071-HTP*

⁎ EE071-HTP protected with solar radiation shielding without mechanical ventilation.

⁎⁎ EE071-HTP protected with solar radiation shielding with mechanical ventilation.

**Fig. 16:**
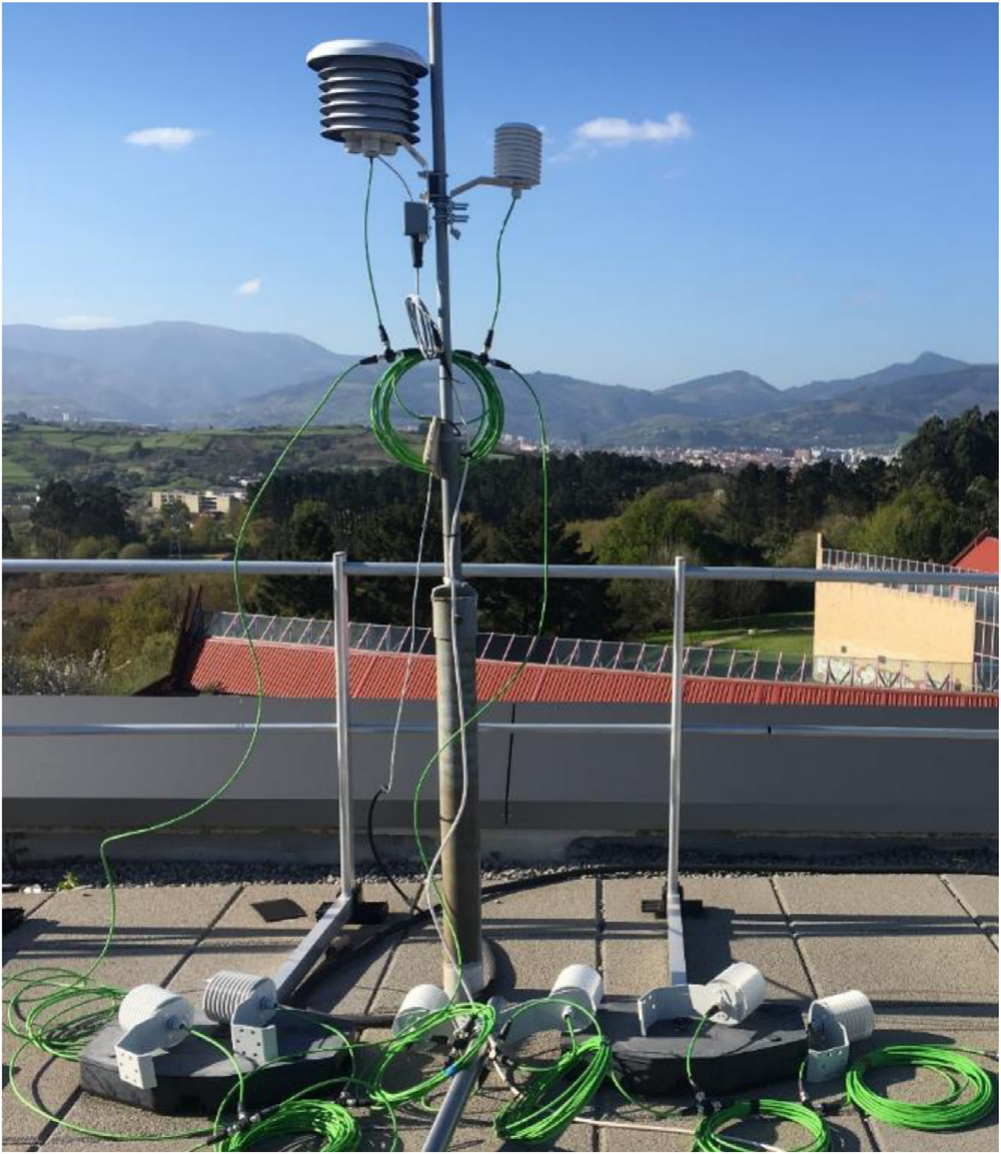
All sensors together test for the exterior 3D Monitoring System (MS).

The exterior experimental test is composed of two tests:

• **Exterior (E) test:** The E test period datasets are prefixed by *E.Fn* and *E.R3*, with *n* = 1 or 2 (seeTable 2).
• **Exterior Together (ET) test:** The ET test period datasets are prefixed by *ET.R3*. All sensors are installed at the same location, five (sensor IDs 20 to 24) over the roof floor, while two (sensor IDs 25 to 26) are on the roof mast (see Fig. 16). The sensor ID 27 is also on a roof mast, but is not shown in Fig. 16.

## Declaration of Competing Interest

The authors declare that they have no known competing financial interests or personal relationships, which have, or could be perceived to have, influenced the work reported in this article.

## Figures and Tables

**Table A1:** .

Acronym	Meaning
A2PBEER	Affordable and Adaptable Public Buildings through Energy Efficient Retrofitting
BAS	Building Automation System
C or °C	Degrees Celsius
CO_2_	Carbon dioxide
e	East
E	Exterior
EPB	Energy Performance Building
ET	Exterior Together test
F	Façade
F0	Ground Floor
F1	Floor One
F2	Floor Two
F3	Floor Three
H	Height Location
ID#	ID Sensor
m2 or m^2^	Square meter
m^3^	Cubic meters
MMS	Mobile Monitoring System
MS	Monitoring System
n	North
OT#	Office Typology Test
OT	Office Typology
pH	Power from heating system
pw	Electricity Power
R	Roof
rad	Radiation
s	South
SI	International System Unit
T#	Tripod Reference
T	Temperature
TCP/IP	Transmission Control Protocol/Internet Protocol
Trad	Radiant Temperature
TT	Tripod Together / Tripod Together Test
UPV/EHU	University of Basque Country
XY	Sensor Measurement
W-m2	Watt per square meter
W/m2	
W/m^2^	
w#	Week of Year
W	Watts
w	West
WS	Workspace

## Appendix A

Table A1

## Appendix B Supplementary materials

Image, application 1

## Footnotes

1 https://en.wikipedia.org/wiki/Internet_protocol_suite
