# Supplementary material for: Dataset of an in-use tertiary building collected from a detailed 3D mobile monitoring system and building automation system for indoor and outdoor air temperature analysis
Source: Data Brief. 2020 Jun 23;31:105907. doi: 10.1016/j.dib.2020.105907 (PMC7341348; doi:10.1016/j.dib.2020.105907)
Supplement: Supplementary file 1 [file mmc1.docx]

**CRediT author statement of Data in Brief:**

**Dataset of an in-use tertiary building collected from a detailed 3D Mobile Monitoring System and Building Automation System for indoor and outdoor air temperature analysis**

**Catalina Giraldo-Soto:** Conceptualization, Methodology, Software, Validation, Investigation, Resources, Data Curation, Writing - Original Draft, Visualization **Aitor Ekoreka:** Conceptualization, Methodology, Validation, Writing - Review & Editing, Supervision, Project administration, Funding acquisition **Ander Barragan:** Methodology, Investigation, Resources **Laurent Mora:** Conceptualization, Methodology, Writing - Review & Editing.
